# Isolation and Characterization of Nickel-Resistant *Microbacterium algeriense* C14 with Plant-Growth-Promoting Properties and Metal-Immobilization Capacity

**DOI:** 10.3390/microorganisms14040875

**Published:** 2026-04-13

**Authors:** Hansheng Liu, Shengxu Wang, Jie Wang, Xingyu Ma, Chunli Zhao, Mingtang Li

**Affiliations:** 1 College of Resources and Environment, Jilin Agricultural University, Changchun 130118, China; 2 College of Forestry and Grassland Science, Jilin Agricultural University, Changchun 130118, China; 3 College of Engineering and Technology, Jilin Agricultural University, Changchun 130118, China

**Keywords:** *Microbacterium algeriense*, nickel resistance, carboxylate precipitation, plant-growth-promoting rhizobacteria, phytoremediation, bioremediation, rhizosphere, heavy metal

## Abstract

Nickel (Ni) contamination threatens plant growth and ecosystem stability, and plant-growth-promoting rhizobacteria (PGPR) are sustainable bioremediation candidates. Here, we isolated and characterized a Ni-resistant PGPR strain, *Microbacterium algeriense* C14, from the rhizosphere of *Zinnia elegans* in Ni-contaminated soil. C14 exhibited exceptional Ni tolerance (up to 800 mg·L^−1^), produced indole-3-acetic acid (IAA), and maintained pH homeostasis (8.3–8.7). XPS and XRD analyses confirmed a novel carboxylate-based precipitation mechanism: C14 secretes carboxyl-containing metabolites that coordinate with Ni^2+^ to form stable amorphous nickel–carboxylate complexes. Under Ni stress (50–600 mg·L^−1^ for germination; 50–600 mg·kg^−1^ soil for pot experiments), C14 inoculation increased the seed germination index by up to 47.3%, seedling root length by 36.9%, and mature plant aboveground fresh weight by 21.32%, while reducing plant Ni uptake by 38.7% (seedlings) and 49.9% (mature shoots). It also enhanced plant antioxidant-enzyme (SOD and POD) activities and soluble protein content, improved soil quality (pH +0.16–0.33 units, urease/acid phosphatase activities elevated), and reduced soil-available Ni by 23.7%. Additionally, C14 enriched Proteobacteria in the rhizosphere and modified microbial community structure. These results highlight *M. algeriense* C14 as a promising resource for Ni-contaminated soil remediation via integrated metal immobilization, growth promotion, and rhizosphere regulation.

## 1. Introduction

Driven by the rapid development of industrialization, mining activities, and intensive agriculture, soil heavy-metal contamination has become a pressing global environmental issue [1,2,3,4,5]. Among various heavy metals, nickel (Ni) pollution has attracted significant attention due to its widespread distribution and profound ecological impacts [6,7]. While Ni serves as an essential micronutrient for plants at low concentrations (1–5 mg kg^−1^), excessive Ni accumulation induces severe phytotoxicity, impairing seed germination, root development, photosynthesis, and overall plant growth [8,9]. Global surveys have documented alarming levels of Ni contamination in industrial and mining regions, where soil Ni concentrations frequently exceed 200 mg kg^−1^ [10,11].

Excessive Ni disrupts soil physicochemical balance, reduces fertility, and interferes with the activity of key soil enzymes (e.g., urease, dehydrogenase, and catalase), thereby hindering soil nutrient cycling processes [12,13]. The bioavailability of Ni is strongly regulated by factors such as soil pH, organic-matter content, and redox potential, which in turn affect its migration risk in the food chain [14]. At the plant level, Ni toxicity manifests as inhibited seed germination, hindered root growth, and reduced biomass, inducing oxidative stress by generating reactive oxygen species (ROS) that damage cell membrane structures. Concurrently, it competitively inhibits the absorption of essential nutrients like Fe, Zn, and Cu by plants, leading to chlorosis, decreased photosynthetic efficiency, and metabolic disorders [15,16,17,18]. Crucially, Ni contamination exerts significant selective pressure on soil microbial communities, where high concentrations of Ni can reduce microbial biomass, alter community structure, and inhibit the growth of sensitive microbial groups [19].

Conventional remediation strategies, such as soil excavation, chemical washing, and amendment-based stabilization, are generally associated with high costs, environmental disruption, and susceptibility to secondary pollution [20,21,22,23]. In contrast, bioremediation—particularly plant–microbe synergistic approaches—has emerged as a promising sustainable alternative, offering both ecological and economic advantages [24,25,26,27]. Plant-growth-promoting rhizobacteria (PGPR) play a central role in alleviating heavy-metal stress through multiple mechanisms: reducing metal bioavailability via precipitation, chelation, or pH modification; promoting plant growth by synthesizing phytohormones; enhancing nutrient acquisition efficiency; and regulating plant physiological responses [28,29,30,31].

Currently, most PGPR research focuses on well-characterized genera, including *Pseudomonas*, *Bacillus*, and *Burkholderia* [32,33,34]; however, these strains exhibit inherent limitations in Ni-contaminated environments: relatively low tolerance to high Ni concentrations (typically 200–600 mg·L^−1^), reliance on external chemical supplements (e.g., urea or phosphate) for efficient metal immobilization, and unstable performance under fluctuating soil pH conditions. By comparison, understudied taxa such as *Microbacterium* demonstrate exceptional metal resistance and unique metabolic traits, yet their Ni detoxification mechanisms and plant-growth-promoting functions remain insufficiently characterized. From the perspective of microbial metal-immobilization mechanisms, existing technologies include biosorption, bioaccumulation, biotransformation, and biomineralization [35,36]. Among these, biomineralization technologies mediated by microbially induced carbonate precipitation (MICP) and microbially induced phosphate precipitation (MIPP) have received considerable attention, but they also require exogenous supplementation of substances like calcium, urea, or phosphate—this not only increases remediation costs but also poses risks of secondary environmental issues such as soil alkalization and eutrophication. This study, thus, isolates and characterizes Ni-resistant PGPR from *Microbacterium*, exploring novel remediation approaches without external chemical additions.

To address these knowledge gaps, this study aimed to isolate and characterize Ni-resistant PGPR from *Zinnia elegans* rhizosphere in Ni-contaminated soil, with the following specific objectives: (1) to identify the core plant-growth-promoting traits (Ni resistance, indole-3-acetic acid (IAA) production, and pH regulation) and elucidate the mechanisms of bacterial-mediated Ni immobilization using X-ray photoelectron spectroscopy (XPS) and X-ray diffraction (XRD); (2) to evaluate the PGPR inoculant’s effects on *Z. elegans* growth performance (from germination to maturity), physiological responses (antioxidant enzymes and soluble protein), and Ni uptake under gradient Ni stress; (3) to assess PGPR-induced changes in soil physicochemical properties, Ni fractions, and rhizosphere microbial community structure. We hypothesized that the selected PGPR would reduce Ni bioavailability through metal precipitation and pH modulation, thereby mitigating plant Ni stress and promoting growth under high Ni contamination. The findings are expected to provide a valuable microbial resource and mechanistic insights for developing efficient, sustainable bioremediation strategies for Ni-contaminated agricultural soils.

## 2. Materials and Methods

### 2.1. Sample Collection and Soil Basic Properties

Soil samples were collected from six representative locations in Hongqiling Town, Jilin Province, China (42°54′ N, 126°25′ E), a region with a history of nickel mining and processing. Sampling sites covered diverse land uses, including agricultural settings (farmer greenhouses, agricultural fields, and farmland streams), natural riparian areas (riverbanks), urban vegetation zones (urban green spaces), and industrial proximate areas (in the vicinity of a nickel factory). A total of 18 Asteraceae plant species were identified across these sites, with *Zinnia elegans* as the dominant ornamental species in urban green spaces. Rhizosphere soil samples were collected from 18 healthy, vigorously growing Asteraceae plant species, placed in sterile bags, and immediately transported to the laboratory.

### 2.2. Isolation, Screening, and Identification of Bacteria

Ten grams of rhizosphere soil was suspended in 90 mL of sterile distilled water and shaken at 160 rpm and 30 °C for 2 h. The supernatant was serially diluted (10^−1^–10^−6^) and inoculated into Luria–Bertani (LB) medium (10 g·L^−1^ tryptone, 5 g·L^−1^ yeast extract, 10 g·L^−1^ NaCl, pH 7.0) supplemented with 400 mg·L^−1^ Ni^2+^ (NiCl_2_·6H_2_O, Sinopharm, Beijing, China) and 60 mg·L^−1^ Cd^2+^ (CdCl_2_·2.5H_2_O, Sinopharm, Beijing, China) for selection. After 3 days of incubation (30 °C, 160 rpm), cultures were spread on LB agar (15 g·L^−1^ agar) with the same Ni^2+^ concentration. Distinct single colonies were purified by repeated streaking (≥3 times) and preserved at 4 °C (short-term) in metal-supplemented LB medium or at −80 °C (long-term) with 30% glycerol.

A total of 121 isolates were screened for plant-growth-promoting (PGP) traits using established methods with three biological replicates per assay. Nitrogen-fixation capacity was assessed by inoculating bacterial suspensions on Ashby’s agar medium (mannitol 10 g·L^−1^ (Sinopharm, Beijing, China), KH_2_PO_4_ 0.2 g·L^−1^ (Sinopharm, Beijing, China), MgSO_4_·7H_2_O 0.2 g·L^−1^ (Sinopharm, Beijing, China), NaCl 0.2 g·L^−1^ (Sinopharm, Beijing, China), K_2_SO_4_ 0.1 g·L^−1^ (Sinopharm, Beijing, China), CaCO_3_ 5.0 g·L^−1^ (Sinopharm, Beijing, China), agar 15 g·L^−1^ (Sinopharm, Beijing, China), pH 7.0) and observing visible colony growth after 5–7 days incubation at 30 °C [37]. Phosphate solubilization ability was evaluated on Pikovskaya’s agar medium (glucose 10 g·L^−1^ (Sinopharm, Beijing, China), Ca_3_(PO_4_)_2_ 5.0 g·L^−1^ (Sinopharm, Beijing, China), (NH_4_)_2_SO_4_ 0.5 g·L^−1^ (Sinopharm, Beijing, China), NaCl 0.2 g·L^−1^, MgSO_4_·7H_2_O 0.1 g·L^−1^, KCl 0.2 g·L^−1^ (Sinopharm, Beijing, China), yeast extract 0.5 g·L^−1^ (Sinopharm, Beijing, China), MnSO_4_·H_2_O 0.002 g·L^−1^ (Sinopharm, Beijing, China), FeSO_4_·7H_2_O 0.002 g·L^−1^ (Sinopharm, Beijing, China), agar 15 g·L^−1^, pH 7.0); strains forming clear halos around colonies after 5 days at 30 °C were considered positive [38]. Potassium solubilization was tested on Aleksandrov agar medium (glucose 5.0 g·L^−1^, MgSO_4_·7H_2_O 0.5 g·L^−1^, CaCO_3_ 0.1 g·L^−1^, FeCl_3_ 0.006 g·L^−1^ (Sinopharm, Beijing, China), Ca_3_(PO_4_)_2_ 2.0 g·L^−1^, potassium feldspar powder 3.0 g·L^−1^ (Sinopharm, Beijing, China), agar 15 g·L^−1^, pH 7.0), with positive strains identified by clear zone formation after 5–7 days at 30 °C [39]. IAA production was quantified by inoculating bacterial cultures in LB broth supplemented with 100 mg·L^−1^ L-tryptophan (Sinopharm, Beijing, China), incubating at 30 °C for 48 h, centrifuging (10,000× *g*, 10 min), and mixing the supernatant with Salkowski reagent (12 g·L^−1^ FeCl_3_ in 7.9 M H_2_SO_4_ (Sinopharm, Beijing, China)) at a 1:2 ratio; after 30 min of dark incubation, absorbance at 530 nm was measured, and IAA concentration was calculated using a standard curve [40]. Siderophore production was detected on Chrome Azurol S (CAS, Sinopharm, Beijing, China) agar plates; bacterial cultures were spot-inoculated and incubated at 30 °C for 5–7 days, with orange halos around colonies indicating positive siderophore activity [41]. ACC deaminase activity was assayed by growing bacteria in DF minimal medium supplemented with 3.0 mM ACC (Sinopharm, Beijing, China) as the sole nitrogen source; after 72 h incubation at 30 °C, bacterial protein content was determined by the Bradford method, and ACC deaminase activity was measured by quantifying α-ketobutyrate production using the ninhydrin method at 540 nm, with activity expressed as nmol α-ketobutyrate mg^−1^ protein h^−1^ [42]. Metal-mineralization capacity was qualitatively assessed by observing visible precipitate formation after the addition of 2 mL of 10 mol·L^−1^ NiCl_2_ solution when bacterial cultures were incubated in LB broth containing 400 mg·L^−1^ Ni^2+^ for 48 h at 30 °C.

Genomic DNA of strain C14 was extracted using a bacterial genomic DNA extraction kit following the manufacturer’s protocol. The 16S rRNA gene was amplified with universal primers 27F (5′-AGAGTTTGATCCTGGCTCAG-3′) and 1492R (5′-GGTTACCTTGTTACGACTT-3′). The 50 μL PCR reaction system included 2× TsingKE Master Mix, 10 μM primers, DNA, and sterile ddH2O. Thermal cycling conditions were: initial denaturation at 94 °C for 10 min; 30 cycles of 94 °C (30 s), 55 °C (30 s), and 72 °C (90 s); a final extension at 72 °C for 10 min. PCR products were purified and sequenced by Allwegene Tech. Co., Ltd. (Beijing, China).

The obtained 16S rRNA gene sequence was compared with GenBank reference sequences via BLAST (version 2.14.0), and a neighbor-joining phylogenetic tree was constructed with MEGA 11.0.

### 2.3. Determination of Bacterial Functional Traits

Growth curves of strain C14 were determined at Ni^2+^ concentrations of 0, 50, 200, 400, 800, and 1200 mg·L^−1^ in LB medium. Cultures (OD_600_ = 0.1) were inoculated into fresh LB medium with the above Ni^2+^ concentrations, incubated at 30 °C with shaking (160 rpm), and OD600 was measured every 6 h for 48 h using a spectrophotometer (752N(PLUS), INESA, Shanghai, China).

Lethal rates were calculated at 6 and 12 h for Ni^2+^ concentrations of 50–2400 mg·L^−1^. Bacterial suspensions (OD600 = 0.5) were exposed to different Ni^2+^ concentrations, and viable cell counts were determined by serial dilution plating. Lethal rate (%) = [(CFU_0_ − CFUₜ)/CFU_0_] × 100, where CFU_0_ and CFUₜ are colony-forming units at time 0 and time t (6 h or 12 h), respectively.

The effect of pH (6, 7, 8, 9, or 10) on bacterial growth and fermentation-broth pH was monitored every 6 h for 36 h. IAA production under different Ni^2+^ concentrations (0–800 mg·L^−1^) was quantified by the Salkowski method after 48 h of incubation. All tests included three biological replicates.

The removal efficiency of different fermentation components (whole broth, cell-free supernatant, washed bacterial cells) for 100 mg·L^−1^ Ni^2+^ was determined by atomic absorption spectroscopy (AAS) after 24 h incubation. Removal efficiency (%) = [(C_0_ − C)/C_0_] × 100, with three biological replicates [43].

To characterize the nickel precipitation mechanism, cell-free fermentation broth was obtained by centrifugation (8000× *g*, 10 min, 4 °C) and filtration through 0.22 μm membranes. Nickel precipitates were generated by adding 50 mL of 10 mol·L^−1^ NiCl_2_ solution to 1 L of cell-free broth, collected by vacuum filtration, washed with deionized water, and dried at 60 °C for 24 h. XPS analysis was performed with an ESCALAB 250Xi spectrometer (Thermo Fisher Scientific, Waltham, MA, USA) using an Al Kα source, and XRD patterns were recorded on a Rigaku MiniFlex 600 diffractometer (Rigaku, Tokyo, Japan) with Cu Kα radiation (θ = 10–80°).

### 2.4. Plant and Soil Experiments

To explore the response of strain C14 to zinnia under nickel stress, two stages of experiments were set up: first, a Petri dish experiment during the seed and seedling stages, and second, a pot experiment during the established seedling stage. Twelve treatments were arranged for the plant and soil experiments: CK: no nickel; CKJ: no nickel/C14; N1: 50 mg·kg^−1^ nickel; N1J: 50 mg·kg^−1^ nickel/C14; N2: 100 mg·kg^−1^ nickel; N2J: 100 mg·kg^−1^ nickel/C14; N3: 200 mg·kg^−1^ nickel; N3J: 200 mg·kg^−1^ nickel/C14; N4: 400 mg·kg^−1^ nickel; N4J: 400 mg·kg^−1^ nickel/C14; N5: 600 mg·kg^−1^ nickel; N5J: 600 mg·kg^−1^ nickel/C14.

*Z. elegans* seeds (thousand-grain weight: 9.5473 g; origin: Xishuangbanna Dai Autonomous Prefecture, China) were surface-sterilized with 75% (*v*/*v*) ethanol for 2 min, and rinsed five times with sterile distilled water. Thirty seeds per treatment were placed in 90 mm sterile Petri dishes with double-layer filter paper, moistened with 10 mL of test solution.

Six Ni^2+^ treatments (0, 50, 100, 200, 400, and 600 mg·L^−1^) were set, with bacterial treatments receiving an additional 0.5 mL of C14 suspension (OD_600_ = 0.8), and controls receiving an equal volume of sterile water. Petri dishes were sealed with Parafilm and incubated in a climate chamber (RXZ-600B, Ningbo Jiangnan Instrument Factory, Ningbo, China) at 19 ± 4 °C, 50–90% relative humidity (24 h darkness followed by a 14 h photoperiod) for 8 days. Germination parameters (germination rate, germination energy, germination index, and vigor index) were calculated, and seedling stem length, root length, and root morphology (WinRHIZO 2016 software) were measured. At harvest, 0.1 g of fresh leaf samples (cotyledons) were collected for physiological analyses: soluble protein (SP, Coomassie Brilliant Blue G250 Method), malondialdehyde (MDA, TBA method), superoxide dismutase (SOD, NBT photoreduction method), and peroxidase (POD, guaiacol oxidation method). Plant nickel content was determined by AAS after HNO_3_-HClO_4_-H_2_O_2_ digestion. Each treatment had three biological replicates.

Pot experiment soil was prepared by mixing garden soil and sand (3:1, *w*/*w*), sieved through a 2 mm mesh. Ni contamination was simulated with NiCl_2_·6H_2_O to final concentrations of 0, 50, 100, 200, 400, and 600 mg kg^−1^ dry soil, adjusted to 60% water-holding capacity, and aged for 30 days (mixed weekly for metal stabilization).

Uniform *Z. elegans* seedlings (2–4 true leaves) were transplanted into plastic pots (20 cm × 18 cm, diameter × height) containing 1.5 kg of aged soil (3 seedlings per pot). Bacterial treatments received 5 mL of C14 suspension at the root zone on day 0 and day 25, with controls receiving sterile water. Plants were grown in a greenhouse, with pots randomly arranged and rotated weekly. Deionized water was added every 2–4 days to maintain 60–70% field capacity; no fertilizers were applied.

Plants were harvested 50 days post-transplantation. Plant height, stem diameter, fresh weights of shoots and roots, and the root–shoot ratio were measured. At harvest, 0.5 g of fresh leaf samples (second fully expanded leaves) were collected for physiological analyses: soluble protein (Coomassie Brilliant Blue G250 Method) [43], malondialdehyde (MDA, TBA method) [44], SOD (NBT photoreduction method) [45], and POD (guaiacol oxidation method) [46]. Plant nickel content was determined by AAS after HNO_3_-HClO_4_-H_2_O_2_ digestion. Each treatment included three biological replicates.

Rhizosphere soil (within 2 mm of roots) was collected at harvest by brushing tightly bound soil after shaking off loose fractions. Soil samples from three plants per pot were pooled to form one composite sample per biological replicate.

Soil moisture content was determined gravimetrically by oven-drying at 105 °C for 24 h. Soil pH and EC were measured in 1:2.5 and 1:5 (*w*/*v*) soil–water suspensions, respectively, after shaking at 200 rpm for 30 min and settling for 30 min. Activities of four soil enzymes (β-glucosidase, urease, acid phosphatase, and polyphenol oxidase) were assayed using ELISA kits (Jianglai Biotechnology, Shanghai, China) following the manufacturer’s protocols.

Total soil nickel was determined after digestion of 0.5 g of samples in Teflon vessels with HNO_3_-HClO_4_-HF (5:1:1, *v*/*v*/*v*). Available nickel was extracted using DTPA solution (0.005 M DTPA + 0.01 M CaCl_2_ + 0.1 M triethanolamine, pH 7.3) at a 1:5 (*w*/*v*) soil–extractant ratio. Nickel concentrations in digests were measured by flame atomic absorption spectrophotometry (AAS). Nickel concentrations in extracts were measured by inductively coupled plasma mass spectrometry (ICP-MS).

### 2.5. Soil Microbial Community Analysis

To explore rhizosphere microbiome changes, composite samples from eight treatments (CK: no nickel, CKJ: no nickel/+ C14, N1: 50 mg·kg^−1^ nickel, N1J: 50 mg·kg^−1^ nickel/+ C14, N2: 200 mg·kg^−1^ nickel, N2J: 200 mg·kg^−1^ nickel/+ C14, N3: 600 mg·kg^−1^ nickel, and N3J: 600 mg·kg^−1^ nickel/+ C14) were used for 16S rRNA gene sequencing. Total genomic DNA was extracted with the FastDNA SPIN Kit for Soil (MP Biomedicals, Santa Ana, CA, USA), and the V3–V4 regions were amplified with primers 338F (5′-ACTCCTACGGGAGGCAGCAG-3′) and 806R (5′-GGACTACHVGGGTWTCTAAT-3′). Amplicons were sequenced on an Illumina MiSeq platform (2 × 300 bp paired-end) by Allwegene Tech. Co., Ltd. (Beijing, China). Sequences were processed to generate OTUs (97% similarity), and α-diversity indices and the taxonomic composition was analyzed. Note: Results are preliminary (*n* = 1 per treatment) and hypothesis-generating.

### 2.6. Statistical Analysis

Data were presented as means ± SD (*n* = 3 biological replicates). Normality (Shapiro–Wilk test) and variance homogeneity (Levene’s test) were verified. Normally distributed data with homogeneous variance were analyzed via one-way ANOVA followed by Duncan’s test (*p* < 0.05/0.01); non-normal data used the Kruskal–Wallis test with Dunn’s post hoc test (Benjamini–Hochberg correction). The majority of analyses were done in SPSS 20.0 (IBM). Spearman’s rank correlation analysis was performed with R 4.4.0 (psych package 2.1.9), and correlation matrices were visualized via corrplot 0.92 (AOE ordering method). Figures were prepared in Origin 2021 and R ggplot2 3.3.5. Microbiome data were descriptively presented without statistical comparisons due to limited replication.

## 3. Results

### 3.1. Isolation, Identification, and Core Functional Characteristics of Strain C14

**Figure 1:**
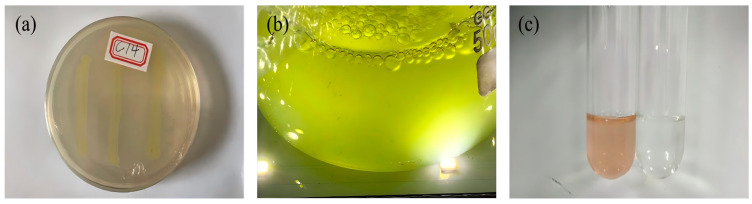
Verification diagrams of colony morphology and function of strain C14: (**a**) colony morphology; (**b**) Ni precipitation product; (**c**) IAA assay.

**Figure 2:**
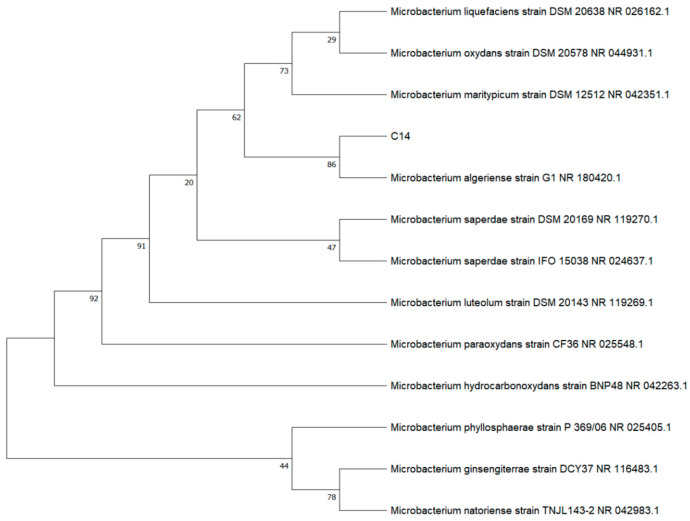
Phylogenetic tree of strain C14 based on 16S rRNA gene sequence.

From the rhizosphere soil of Asteraceae plants collected at six representative sites in Hongqiling Town, 121 heavy-metal-resistant strains were isolated using LB medium supplemented with high concentrations of Ni^2+^ (400 mg·L^−1^). Among them, seven strains showed high resistance, and at least one plant-growth-promoting (PGP) trait. Strain C14, isolated from the rhizosphere of *Zinnia elegans*, was selected as the target strain due to its unique combination of metal mineralization capacity and IAA production, which distinguished it from other single-function strains. Figure 1 displays the functional verification diagram of the colony morphology, Ni precipitation product, and IAA assay of strain C14. Colonies of C14, cultured on LB agar at 28 °C for 48 h, are yellowish-white, with smooth surfaces, neat edges, and convex elevations. 16S rRNA gene sequencing revealed that C14 shares 100% homology with *Microbacterium algeriense* (GenBank: NR 180420), confirming its taxonomic status as *Microbacterium algeriense* C14 (GenBank: PX 488027). To further confirm its taxonomic status, a phylogenetic tree was constructed based on the 16S rRNA sequence of strain C14 and other similar type strains (Figure 2).

**Figure 3:**
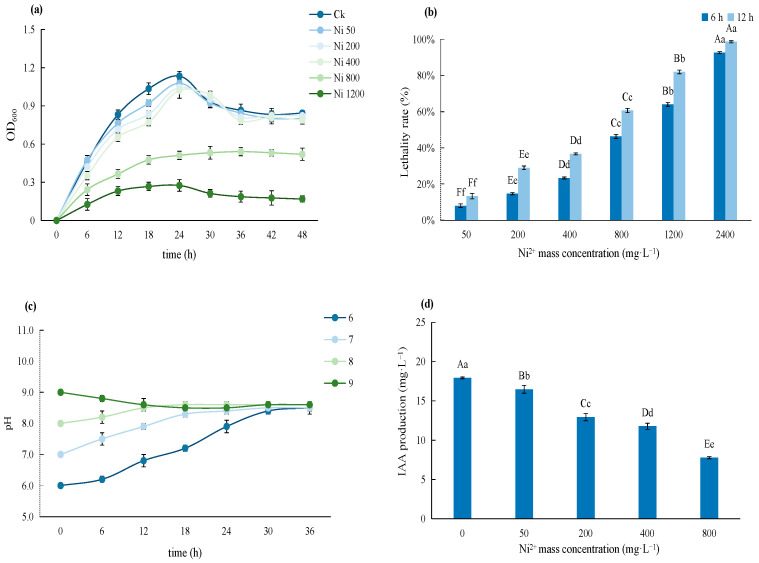
The comprehensive effects of Ni^2+^ on the growth, lethality, IAA synthesis, and pH regulation of strain C14: (**a**) effect of Ni^2+^ concentration on the growth of strain C14; (**b**) effect of Ni^2+^ concentration on the lethality of strain C14 (6 h and 12 h); (**c**) regulatory effect of initial medium pH on the fermentation-broth pH of strain C14; (**d**) effect of Ni^2+^ concentration on IAA synthesis by strain C14.Note: The data in the table are means and standard deviations; different lowercase letters in the same column indicate significant differences (*p* < 0.05), while different uppercase letters in the same column indicate extremely significant differences (*p* < 0.01).

Strain C14 showed exceptional Ni resistance, sustaining viable growth at Ni^2+^ concentrations up to 800 mg·L^−1^ (maximum OD_600_ = 75.5% of the control, 12 h mortality rate = 68.7 ± 4.5%; Figure 3). It adapted to weakly acidic to alkaline environments (pH 6–9), and autonomously regulated fermentation-broth pH to 8.3–8.7. For PGP traits, C14 produced IAA with a maximum yield of 18.5 ± 1.8 μg·mL^−1^ in control medium; even at 800 mg·L^−1^ Ni^2+^, IAA production retained 47% of the control level, ensuring stable growth-promoting capacity under severe stress.

### 3.2. Nickel-Immobilization Mechanism of Strain C14

**Figure 4:**
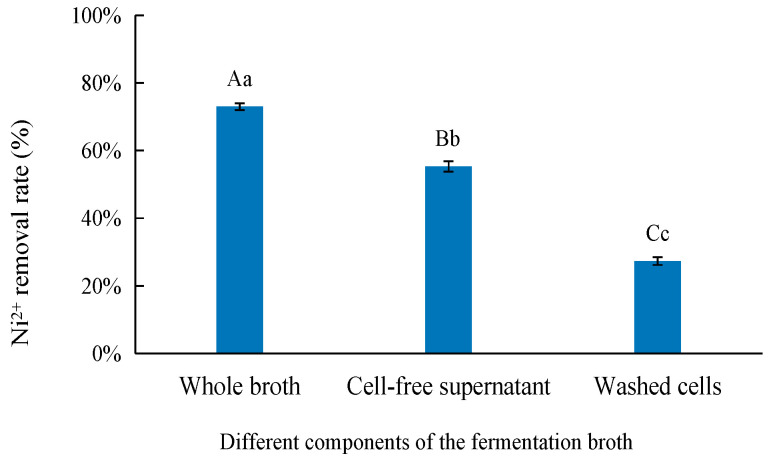
Removal of Ni^2+^ by different fractions of fermentation broth of strain C14. Note: Different lowercase letters in the same figure indicate significant differences (*p* < 0.05), while different uppercase letters in the same figure indicate extremely significant differences (*p* < 0.01).

Strain C14 immobilizes nickel primarily through secreted metabolites rather than cell surface adsorption. Fractionation experiments showed that the cell-free fermentation broth achieves a Ni^2+^ removal rate of 65.3 ± 2.8%, which is significantly higher than that of washed bacterial cells (28.7 ± 2.1%), indicating that carboxyl-containing organic metabolites are the key functional components (Figure 4).

**Figure 5:**
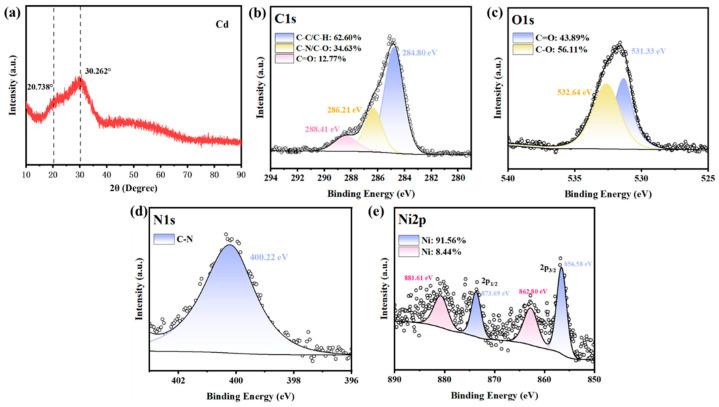
XRD and XPS spectra of the precipitated product: (**a**) XRD; (**b**) C1s; (**c**) O1s; (**d**) N1s; (**e**) Ni2p.

As shown in Figure 5, XPS and XRD analyses clarified the immobilization mechanism: XPS spectra revealed characteristic peaks corresponding to C=O (288.1 eV in C1s, 531.2 eV in O1s) and Ni^2+^ (856.3 eV and 873.8 eV in Ni2p), confirming the coordination between nickel ions and carboxyl groups (-COOH) from bacterial metabolites. XRD patterns showed broad amorphous peaks (Ni precipitate at 2θ = 23.18°), excluding crystalline nickel compounds such as Ni(OH)_2_ or NiCO_3_. Combined with elemental analysis, the precipitates are identified as stable amorphous nickel–carboxylate complexes with the proposed formula R-C_2_H_3_O_2_NNi (R denotes amino-acid residues), formed by coordination between Ni^2+^ and carboxyl groups from bacterial metabolic products. This mechanism requires no exogenous chemical supplements and exhibits stable immobilization effects across a wide pH range.

### 3.3. Effects of Strain C14 on Growth and Physiology of Zinnia elegans

#### 3.3.1. Seed Germination and Seedling Growth

As shown in Table 1, nickel stress exhibits a “low-promotion, high-inhibition” effect on *Zinnia elegans* seed germination. As shown in Table 1, low concentrations (50 mg·L^−1^ Ni^2+)^ slightly stimulate germination (germination index increased by 25.2% compared to the control), while concentrations above 200 mg·L^−1^ significantly inhibit germination parameters. At 600 mg·L^−1^ Ni^2+^, the germination rate and vigor index decrease by 29.5% and 60.0%, respectively, compared to the control. Inoculation with C14 significantly alleviates nickel toxicity across all concentration gradients. At 50 mg·L^−1^ Ni^2+^ (N1J), the germination index and vigor index increase by 47.3% and 58.3%, respectively, compared to the non-inoculated group (N1). Even under severe stress (600 mg·L^−1^ Ni^2+^, N5J), the germination percentage is 55.6%—7.8% higher than that of the non-inoculated group (N5)—and the vigor index is significantly improved.

As shown in Table 2, Nickel stress exerted a distinct dose-dependent inhibitory effect on the seedling growth of *Zinnia elegans*, with all measured growth parameters (stem length, root length, root surface area, root projection area, and root volume) showing significant decreasing trends as the Ni^2+^ concentration increased. For stem length, a slight increase was observed at a low Ni^2+^ concentration of 50 mg·L^−1^ (N1: 6.041 cm) compared with the control (CK: 5.252 cm), while a sharp decline occurred with the further increase in Ni^2+^ concentration, and the stem length was only 3.028 cm at 600 mg·L^−1^ Ni^2+^ (N5), a reduction of 42.3% relative to the control. Root length was the most sensitive parameter to Ni^2+^ stress, plummeting from 4.922 cm in the control (CK) to 0.502 cm at 600 mg·L^−1^ Ni^2+^ (N5), with a decrease of 89.8%. In addition, root surface area, projection area, and volume also decreased remarkably with the elevation of Ni^2+^ concentration; the root volume reduced from 0.011 cm^3^ (CK) to 0.002 cm^3^ (N5), which indicated that Ni^2+^ stress caused severe inhibition of root-system development in *Zinnia elegans* seedlings. Inoculation with *Microbacterium algeriense* C14 significantly alleviated the adverse effects of Ni^2+^ stress on seedling growth across all concentration gradients. Under non-stress conditions (CKJ), C14 inoculation slightly improved all growth indices of *Zinnia elegans* seedlings compared with the non-inoculated control (CK), with moderate increases in stem length, root length, and root volume. At the low Ni^2+^ stress level of 50 mg·L^−1^ (N1J), the seedlings inoculated with C14 exhibited the maximum stem length (6.519 cm) and root length (5.124 cm) among all treatments, which were higher than those of both the non-inoculated group (N1) and the non-stress control (CK). Even under moderate-to-high Ni^2+^ stress, C14 inoculation consistently maintained better growth indices of seedlings than the corresponding non-inoculated groups. For instance, at 600 mg·L^−1^ Ni^2+^ (N5J), the stem length and root length of seedlings were 3.129 cm and 0.687 cm, respectively—both higher than those of the non-inoculated group (N5: 3.028 cm and 0.502 cm). Moreover, C14 inoculation also improved the root surface area, projection area, and volume of seedlings under all Ni^2+^ stress concentrations, which suggested that C14 could effectively promote root development of *Zinnia elegans* under Ni stress, thereby enhancing the potential of seedlings for nutrient and water uptake.

#### 3.3.2. Seedling Physiological Responses

**Figure 6:**
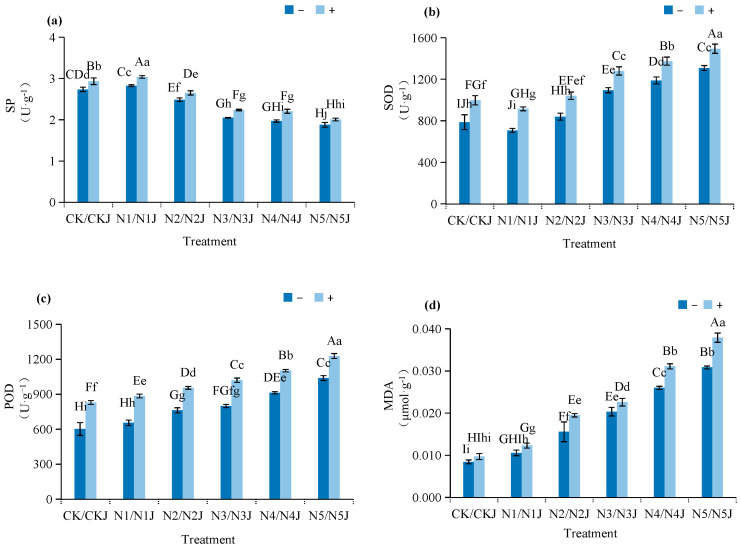
Effect of nickel stress under *Microbacterium algeriense* C14 on physiological indices in *Zinnia elegans* Jacq. seedlings: (**a**) Soluble protein; (**b**) superoxide dismutase; (**c**) peroxidase; (**d**) malondialdehyde. Note: In the figure, different lowercase letters in the same figure indicate significant differences (*p* < 0.05), while different uppercase letters in the same figure indicate extremely significant differences (*p* < 0.01). “−” indicates no application, “+” indicates application.

Nickel stress significantly altered the physiological metabolism of *Zinnia elegans* seedlings, while inoculation with *Microbacterium algeriense* C14 effectively regulated the plant’s stress-response mechanism (Figure 6).

Under nickel stress, the soluble protein (SP) content of *Zinnia elegans* seedlings showed a trend of first increasing and then decreasing, with the peak observed in the N1 treatment (50 mg·L^−1^ Ni^2+^). Compared with the control (CK), the SP content in N1 increased by 3.25%, but decreased by 9.07%, 25.09%, 28.05%, and 31.32% in N2 (100 mg·L^−1^), N3 (200 mg·L^−1^), N4 (400 mg·L^−1^), and N5 (600 mg·L^−1^) treatments, respectively. Inoculation with C14 enhanced the SP content across all nickel concentration gradients: the SP content in the C14-inoculated groups (N1J–N5J) was 6.54–12.01% higher than that in the non-inoculated groups (N1–N5) under the same stress conditions, with N1J (50 mg·L^−1^ Ni^2+^ + C14) showing the highest SP content (7.56% higher than N1), which improved the plant’s osmotic-adjustment ability and intracellular metal-chelation capacity.

For antioxidant-enzyme activities, the superoxide dismutase (SOD) activity of seedlings under nickel stress first decreased and then increased, reaching the maximum in the N5 treatment. Compared with CK, SOD activity decreased by 9.99% in N1 but increased by 6.80%, 39.20%, 51.20%, and 66.40% in N2, N3, N4, and N5, respectively. C14 inoculation further enhanced SOD activity, with N1J–N5J showing significantly higher levels than the corresponding non-inoculated group C14, where SOD activity was 14.18% higher than N5, effectively scavenging reactive oxygen species (ROS) induced by heavy metals. The peroxidase (POD) activity of seedlings showed a gradual upward trend with increasing nickel concentration, peaking in N5. Compared with CK, POD activity increased by 8.75%, 26.69%, 32.78%, 51.61%, and 72.54% in N1–N5, respectively. C14 inoculation further improved POD activity by 18.23–35.13% across all concentration gradients, with N5J showing a 18.23% higher POD activity than N5, enhancing the plant’s ability to decompose hydrogen peroxide and alleviate oxidative damage.

Malondialdehyde (MDA) content, an indicator of lipid peroxidation, showed a gradual increase with increasing nickel concentration, reaching the highest value in N5. Compared with CK, MDA content increased by 25.31%, 84.60%, 141.11%, 208.31%, and 265.62% in N1–N5, respectively. Inoculation with C14 significantly increased MDA accumulation: the MDA content in N1J–N5J was 16.40–25.26% higher than that in the corresponding non-inoculated groups, with N5J showing the most significant increastion (22.92% higher than N5), indicating that C14 may regulate the lipid peroxidation response of Zinnia elegans seedlings under nickel stress; the most significant promotion was in N5J (100 mg·L^−1^ Ni^2+^ + C14).

#### 3.3.3. Nickel Accumulation in Seedlings

**Figure 7:**
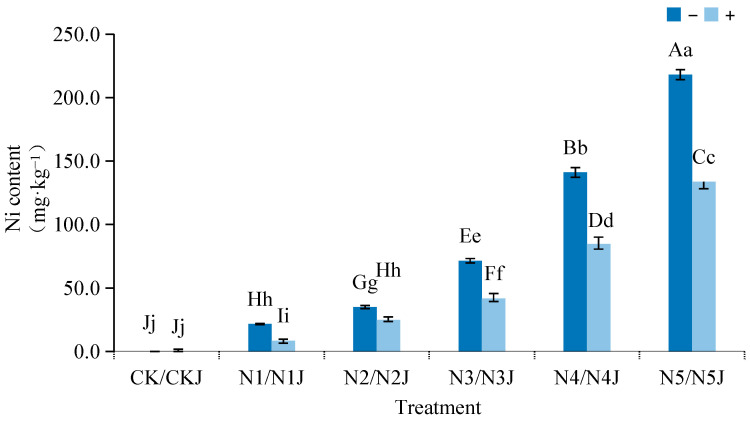
Effect of adding *Microbacterium algeriense* C14 on nickel enrichment in *Zinnia elegans* Jacq. seedlings under nickel stress. Note: In the figure, different lowercase letters in the same figure indicate significant differences (*p* < 0.05), while different uppercase letters in the same figure indicate extremely significant differences (*p* < 0.01). “−” indicates no application, “+” indicates application.

The nickel content in *Zinnia elegans* seedlings showed a significant positive correlation with the external nickel concentration (Figure 7).

Under nickel stress alone, the nickel accumulation in seedlings increased sharply with increasing nickel concentration. Inoculation with *M. algeriense* C14 significantly inhibited nickel uptake by seedlings: across all nickel concentration gradients. For example, at 600 mg·L^−1^ Ni^2+^ (N5J), the nickel content decreased from 218.17 mg·kg^−1^ (N5) to 133.66 mg·kg^−1^. This reduction in plant nickel accumulation is closely related to C14’s carboxylate-based nickel-immobilization mechanism, which reduces soil-available nickel content and limits nickel bioavailability to the plant.

#### 3.3.4. Mature Plant Growth

As shown in Table 3, at the mature stage (50 days after transplanting), high nickel concentrations (600 mg·kg^−1^ soil) significantly inhibit plant growth: plant height, aboveground fresh weight, and underground fresh weight decrease by 39.7%, 45.38%, and 68.64%, respectively, compared to the control. C14 inoculation maintains superior growth performance across all nickel concentrations. At 50 mg·kg^−1^ Ni^2+^ (N1J), plant height, stem width, and aboveground fresh weight reach the maximum, increasing by 3.77%, 2.49%, and 8.73%, respectively, compared to the inoculated control (CKJ). Even at 600 mg·kg^−1^ Ni^2+^ (N5J), aboveground fresh weight and underground fresh weight are 21.32% and 19.82% higher than those of the non-inoculated group, demonstrating sustained growth-promoting effects throughout the plant life cycle.

#### 3.3.5. Physiological Responses

**Figure 8:**
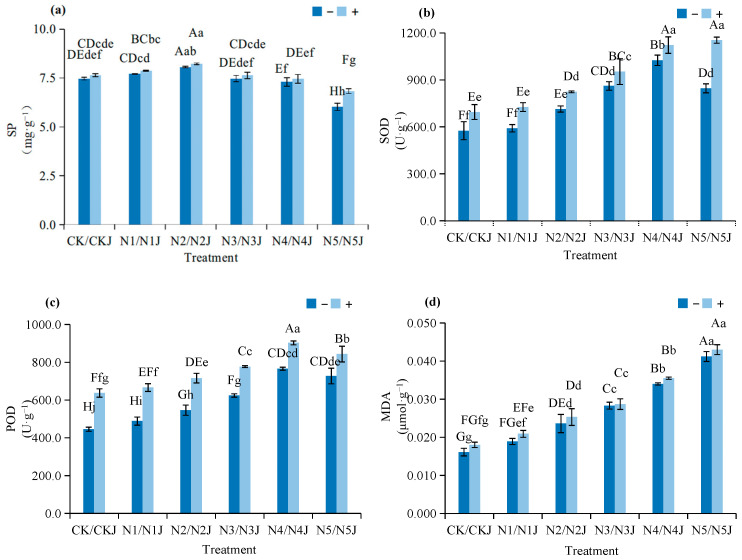
Effect of nickel stress under *Microbacterium algeriense* C14 on physiological indices in *Zinnia elegans* Jacq. mature plants: (**a**) Soluble protein; (**b**) superoxide dismutase; (**c**) peroxidase; (**d**) malondialdehyde. Note: In the figure, different lowercase letters in the same figure indicate significant differences (*p* < 0.05), while different uppercase letters in the same figure indicate extremely significant differences (*p* < 0.01). “−” indicates no application, “+” indicates application.

As shown in Figure 8, C14 inoculation enhances the stress resistance of *Zinnia elegans* by regulating physiological metabolism under nickel stress:

1. Soluble Protein Content: Increased by 2.07–13.27% across concentration gradients, improving osmotic adjustment and intracellular metal-chelation capacity.
2. Antioxidant-Enzyme Activities: SOD activity increases by9.51–36.43% across concentration gradients, enhancing the scavenging capacity of reactive oxygen species (ROS). POD activity increases by 15.95–43.11% across concentration gradients, strengthening the defense ability against oxidative stress.
3. Lipid Peroxidation: MDA content is 1.41–11.80% higher than that of the non-inoculated group under the same stress, which still helps to alleviate membrane damage and maintain cellular integrity.

#### 3.3.6. Nickel Accumulation in Plants

**Figure 9:**
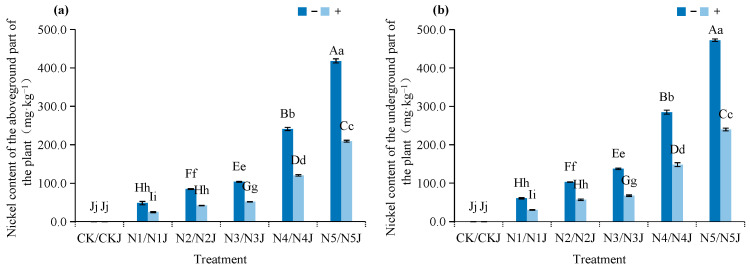
Effect of *Microbacterium algeriense* C14 on the aboveground and underground nickel contents of *Zinnia elegans* Jacq. mature plants formation under nickel stress: (**a**) Above ground; (**b**) underground. Note: In the figure, different lowercase letters in the same figure indicate significant differences (*p* < 0.05), while different uppercase letters in the same figure indicate extremely significant differences (*p* < 0.01). “−” indicates no application, “+” indicates application.

Nickel content in *Zinnia elegans* tissues increases with external nickel concentration, with root nickel content consistently higher than shoot content. As shown in Figure 9, C14 inoculation significantly reduces plant nickel uptake: across concentration gradients, shoot and root nickel contents decrease by 48.22–50.36% and 44.87–50.81%, respectively. At 600 mg·kg^−1^ soil Ni^2+^, shoot nickel content decreases from 418.17 mg·kg^−1^ (non-inoculated) to 209.18 mg·kg^−1^ (inoculated), and root nickel content decreases from 472.30 mg·kg^−1^ to 293.61 mg·kg^−1^, significantly reducing plant metal exposure risk.

### 3.4. Effects of Strain C14 on Soil Environment and Microbial Community

#### 3.4.1. Soil Physicochemical Properties and Enzyme Activities

**Figure 10:**
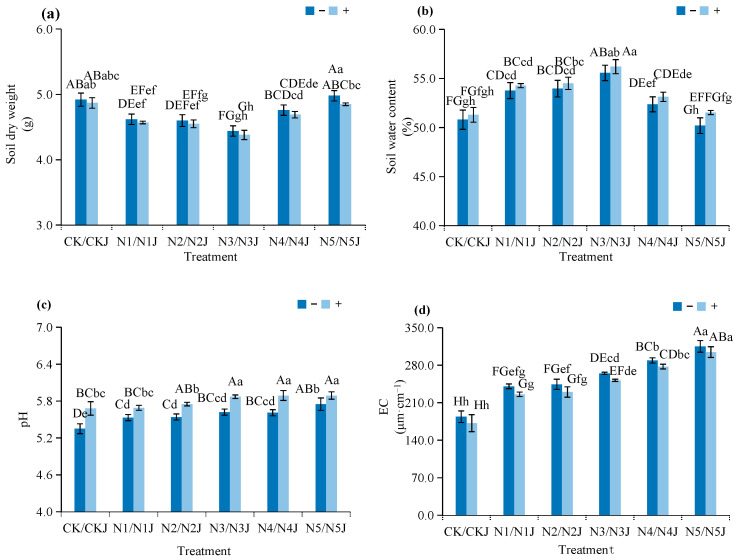
Effects of Microbacterium algeriense strain C14 and Zinnia elegans Jacq. on physical. properties of nickel-contaminated soil: (**a**) Soil dry weight; (**b**) Soil water content; (**c**) Soil pH value; (**d**) S oil electrical conductivity. Note: In the figure, different lowercase letters in the same figure indicate significant differences (*p* < 0.05), while different uppercase letters in the same figure indicate extremely significant differences (*p* < 0.01). “−” indicates no application, “+” indicates application.

**Figure 11:**
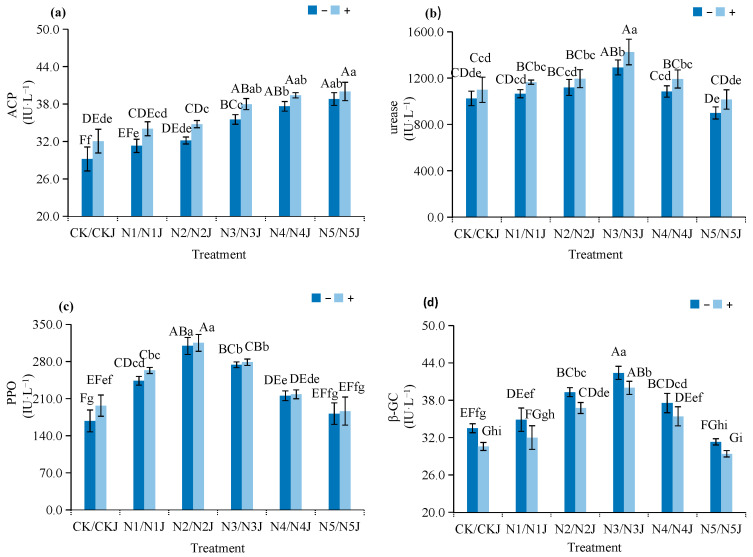
Effects of *Microbacterium algeriense* C14 and *Zinnia elegans* Jacq. on soil enzyme activity indices of nickel-contaminated soil: (**a**) acid phosphatase; (**b**) urease; (**c**) polyphenol oxidase; (**d**) β-glucosidase. Note: In the figure, different lowercase letters in the same figure indicate significant differences (*p* < 0.05), while different uppercase letters in the same figure indicate extremely significant differences (*p* < 0.01). “−” indicates no application, “+” indicates application.

C14 inoculation improves soil environmental quality under nickel stress:

1. Soil pH: Increases by 0.16–0.33 units, shifting the soil towards a weakly alkaline environment that reduces nickel bioavailability (Figure 10).
2. Soil Electrical Conductivity: Decreases by 4.75–6.52% across all nickel concentration gradients, indicating that C14 inoculation can reduce the salt content in the soil, alleviate soil salinization caused by nickel stress, and further optimize the soil physical and chemical environment (Figure 10)
3. Soil Enzyme Activities: Urease and acid phosphatase activities increase by 7.22–10.40% and 6.34–9.74%, respectively, at moderate nickel concentrations (N2–N3 groups), enhancing soil nutrient cycling capacity; in addition, polyphenol oxidase activity is slightly elevated by 1.52–17.21%, while β-glucosidase activity is moderately reduced by 5.68–7.71%, reflecting functional optimization of the soil enzyme system (Figure 11). Specifically, urease and ACP activities reached the highest increase in the N3J group, which was consistent with the optimal growth state of plants under moderate nickel stress.

#### 3.4.2. Soil Nickel Content and Available Fraction

**Figure 12:**
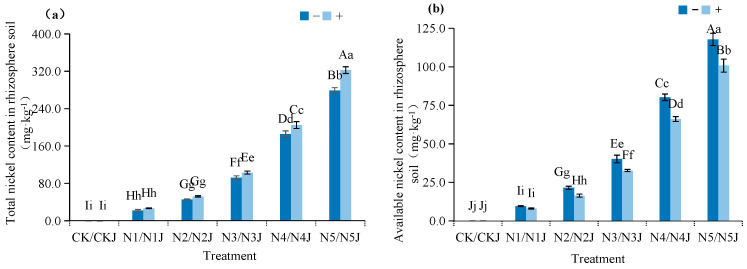
Effect of *Microbacterium algeriense* C14 on the aboveground nickel content of *Zinnia elegans* Jacq. mature plants formed under nickel stress: (**a**) total nickel content in rhizosphere soil; (**b**) available nickel content in rhizosphere soil. Note: In the figure, different lowercase letters in the same figure indicate significant differences (*p* < 0.05), while different uppercase letters in the same figure indicate extremely significant differences (*p* < 0.01). “−” indicates no application, “+” indicates application.

The combined action of strain C14 and *Zinnia elegans* also led to an overall increase in the total nickel content of nickel-contaminated soil under different nickel stress concentrations, with the total rhizosphere soil nickel content reaching a peak of 322.46 mg·kg^−1^ in the N5J treatment; this value was a 57.39% increase relative to the N4J treatment (Figure 12a). The addition of strain C14 significantly elevated the total nickel content in the rhizosphere soil under the same nickel stress concentration, indicating a remarkable immobilization effect and a consequent reduction in nickel uptake by *Zinnia elegans*. The bioavailable nickel content in the rhizosphere soil of *Zinnia elegans* exhibited an overall upward trend with increasing nickel stress concentration, peaking at 117.8 mg·kg^−1^ in the N5 treatment (Figure 12b). Under different nickel stress concentrations, the synergistic effect of strain C14 and *Zinnia elegans* resulted in an overall increase in the bioavailable nickel content of nickel-contaminated soil, with the bioavailable nickel content in the rhizosphere soil reaching a peak of 100.84 mg·kg^−1^ in the N5J treatment. Notably, the addition of strain C14 effectively reduced the bioavailable nickel content in the rhizosphere soil under the same nickel stress concentration, significantly mitigating the risk of nickel stress to the root system of *Zinnia elegans*.

### 3.5. Rhizosphere Microbial Community

Preliminary 16S rRNA sequencing of representative samples (*n* = 1 per treatment) shows that nickel stress and C14 inoculation alter the rhizosphere microbial community structure: under high nickel stress (600 mg·kg^−1^), the non-inoculated group (N3) has a higher Shannon index (9.89) than the control (9.45), while the C14-inoculated group (N3J) shows a reduced Shannon index (6.90), suggesting potential microbiome-mediated mechanisms (preliminary results, Table 4).

**Figure 13:**
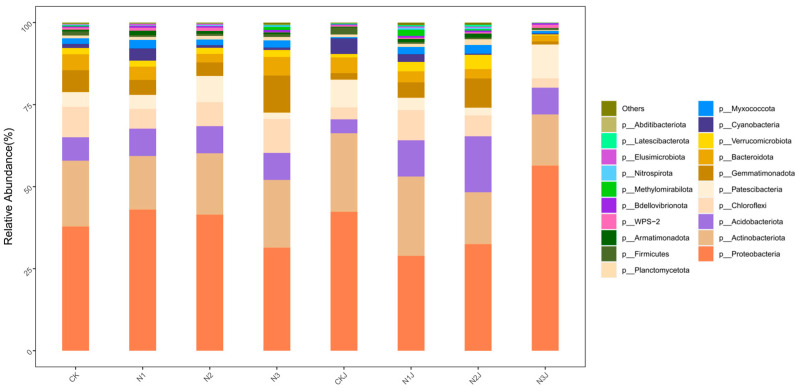
Microbial community structure of all samples under different treatments at the phylum level.

**Figure 14:**
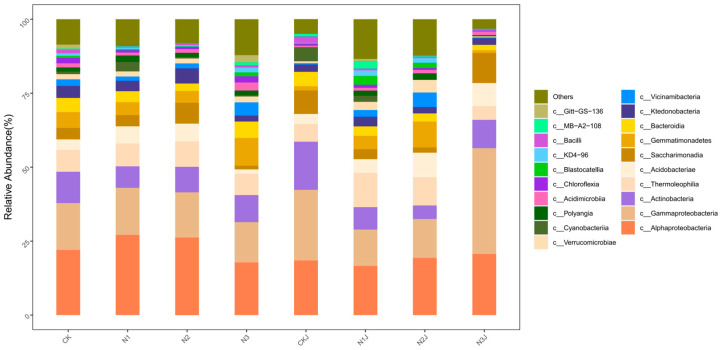
Microbial community structure of all samples under different treatments at the class level.

In this study, bacterial taxa with a relative abundance exceeding 0.1% of the total community at the phylum and class levels were defined as the dominant microbiota. Variations in the proportion and species of dominant microbiota at the phylum and class levels were observed across all treatments following the inoculation of *M. algeriense* C14 (Figure 13 and Figure 14). Overall, the top 10 dominant bacterial phyla across the ten treatments were Proteobacteria, Actinobacteriota, Acidobacteriota, Chloroflexi, Patescibacteria, Gemmatimonadota, Bacteroidota, Myxococcota, and Verrucomicrobiota. Among these, the most abundant phyla were Proteobacteria (48.77% in N3), Actinobacteriota (23.92% in CKJ), Acidobacteriota (13.00% in N2J), and Chloroflexi (8.78% in N2J).

At the class level, the top 10 dominant taxa across all treatments included Alphaproteobacteria, Gammaproteobacteria, Actinobacteria, Thermoleophilia, Saccharimonadia, Ktedonobacteria, Gemmatimonadetes, and Vicinamibacteria. The most prevalent classes were Alphaproteobacteria (27.10% in N1), Gammaproteobacteria (35.72% in N3J), Actinobacteria (16.26% in CKJ), Thermoleophilia (11.59% in N1J), and Saccharimonadia (7.94% in CKJ).

**Figure 15:**
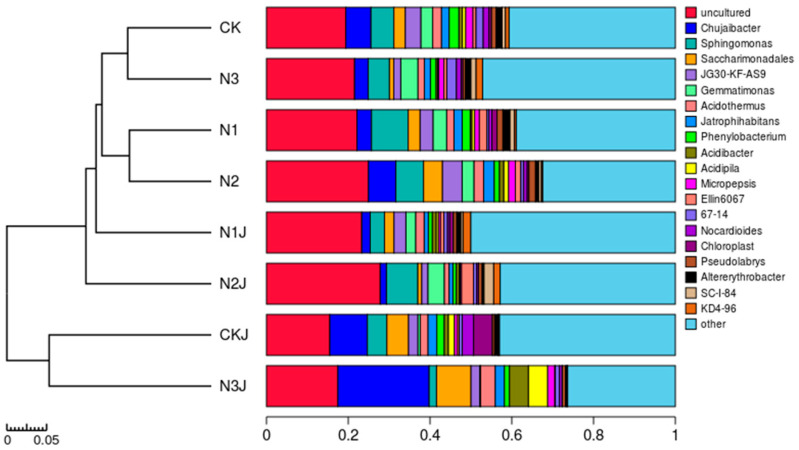
Microbial species structure of all samples at the genus level.

At the genus level, the bacterial sequences were assigned to a total of 762 genera, including both identified and unclassified taxa. Cluster analysis was performed based on the relative abundance of each genus across treatments (Figure 15). The unclassified uncultured genus was dominant in all C14-inoculated treatments, accounting for 15.50–24.94% of the total valid sequences in the samples. All treatments contained a high abundance of core genera, including Sphingomonas, Chujaibacter, Saccharimonadales, JG30-KF-AS9, Acidothermus, Gemmatimonas, Jatrophihabitans, Ellin6067, and Phenylobacterium.

**Figure 16:**
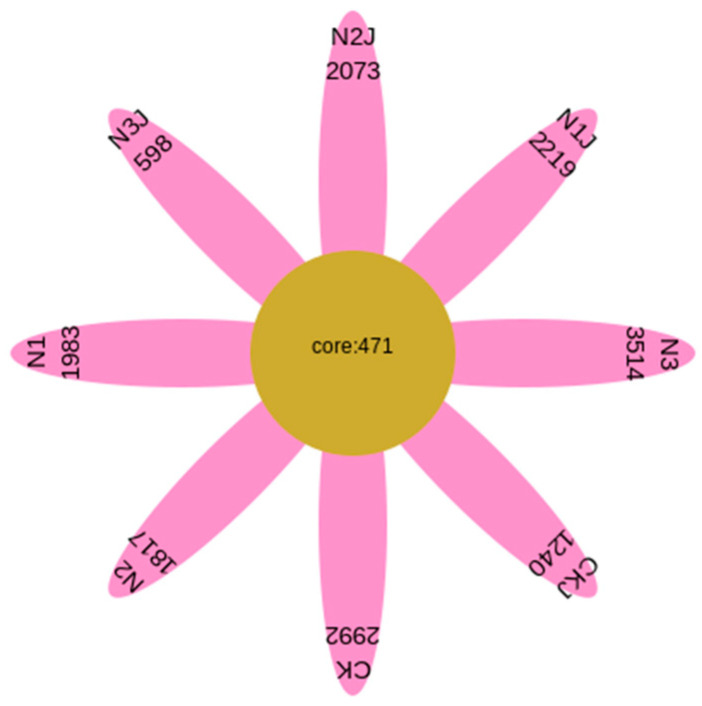
Petal diagram of OTUs within the bacterial community at a cutoff value of 0.03.

A comparison of the shared bacterial OTUs across all treatments (Figure 16) revealed that the number of unique OTUs for CK, N1, N2, N3, CKJ, N1J, N2J, and N3J were 2992, 1983, 1817, 3514, 1112, 1240, 2219, and 598, respectively. The overlapping region indicated the number of OTUs shared among samples, with a total of 471 core OTUs shared across all eight treatments, accounting for 2.33% of the total OTUs. Among all treatments, the N3 group (non-inoculated, 600 mg·kg^−1^ Ni^2+^) had the highest number of OTUs, while the N3J group (C14-inoculated, 600 mg·kg^−1^ Ni^2+^) had the lowest number of OTUs.

### 3.6. Correlation Analysis

**Figure 17:**
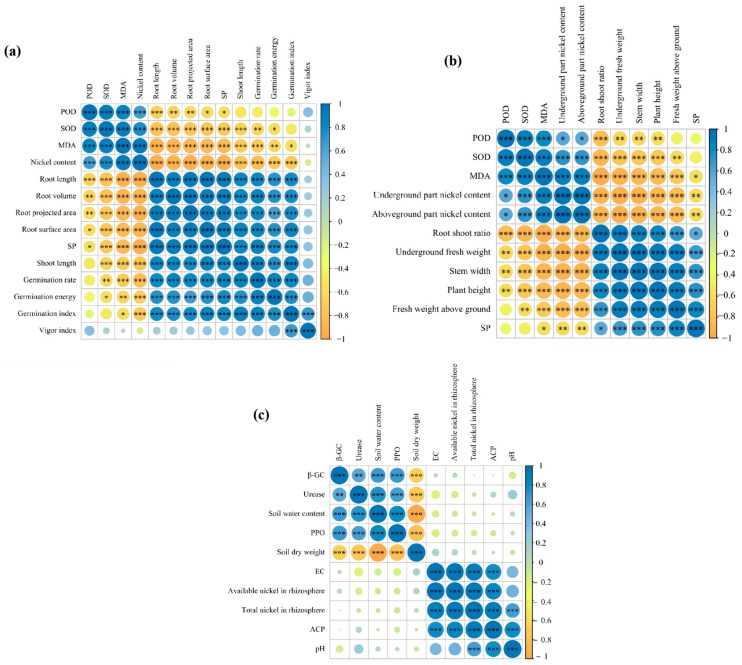
Spearman’s correlation analysis of growth, physiological, and soil indicators of *Zinnia elegans* at the seedling and adult-plant stages under Nickel stress. (**a**) Seedling stage indices; (**b**) mature plant stage indices; (**c**) Soil environmental indices. Note: Asterisks indicate statistical significance: * *p* < 0.05, ** *p* < 0.01, *** *p* < 0.001.

Spearman’s rank correlation analysis revealed distinct clustering patterns among the measured indicators (Figure 17).

Under nickel stress alone, the correlations among indicators exhibited clear clustering characteristics. Peroxidase (POD), superoxide dismutase (SOD), malondialdehyde (MDA), and nickel content were clustered into one group, showing a significant positive correlation. Seedling morphological indicators (root length, root projection area, and root surface area), growth and development indicators (germination rate, germination energy, and germination index), and soluble protein content formed another cluster, displaying a significant positive correlation. A significant negative correlation was observed between the two aforementioned clusters. Additionally, the vigor index was significantly and positively correlated with POD activity, germination rate, germination energy, and germination index.

Under nickel stress alone, the growth and physiological indicators of mature plants were also clustered into two distinct groups. POD, SOD, MDA, root nickel content, and shoot nickel content were grouped together, displaying a significant positive correlation. Growth and development indicators of mature plants (plant height, stem diameter, shoot fresh weight, root fresh weight, and root–shoot ratio) and soluble protein content constituted the second cluster, displaying a significant positive correlation. A significant negative correlation was found between these two clusters of mature plant indicators.

For soil indicators under nickel stress alone, two distinct correlation clusters were identified as well. β-glucosidase activity, urease activity, soil moisture content, polyphenol oxidase activity, and soil dry weight were clustered together: all indicators except soil dry weight showed significant positive correlations with each other, while soil dry weight was significantly and negatively correlated with the other indicators in this cluster. Electrical conductivity, DTPA-extractable nickel content in rhizosphere soil, total nickel content in rhizosphere soil, and acid phosphatase activity formed the second cluster, displaying significant positive correlations among all members. No obvious correlation was detected between these two soil indicator clusters.

## 4. Discussion

### 4.1. Multifunctional Traits of M. algeriense C14

*M. algeriense* C14 is a rare nickel-tolerant PGPR strain with multiple key traits. It exhibits exceptional nickel resistance, tolerating up to 800 mg·L^−1^ Ni^2+^ while maintaining 75.5% of the growth rate of the control group. This resistance level surpasses most commonly studied PGPR genera such as *Pseudomonas* and *Bacillus* [47,48,49]. Even under severe nickel stress, C14 sustains IAA production, retaining 47% of the control group’s yield at 800 mg·L^−1^ Ni^2+^, ensuring stable growth-promoting potential. Additionally, C14 possesses a pH self-regulation capability, actively adjusting the fermentation-broth pH to an optimal range of 8.3–8.7 across initial pH values of 6–9, enhancing its adaptability to diverse soil environments. Nickel contamination exerts multiple toxic effects on soil ecosystems and plant health, including direct inhibition of microbial activity, disruption of nutrient cycling, induction of oxidative stress in plants, and competitive interference with essential nutrient uptake. Strain C14 has evolved integrated countermeasures to cope with these multifaceted challenges: its exceptional nickel tolerance (up to 800 mg·L^−1^) enables survival and metabolic activity in highly contaminated environments where sensitive microorganisms are suppressed; the pH self-regulation capacity (maintaining 8.3–8.7) counteracts nickel-induced soil acidification and maintains optimal conditions for both bacterial function and nutrient availability; sustained IAA production under nickel stress (47% retention at 800 mg·L^−1^) ensures continued growth promotion even when plant hormonal balance is disrupted by metal toxicity; and the carboxylate-based immobilization mechanism directly reduces the bioavailable nickel fraction that causes toxicity. This integrated suite of traits allows C14 to not only survive nickel stress but actively counteract its multiple negative impacts on the plant–soil system.

### 4.2. Novel Carboxylate-Based Nickel-Immobilization Mechanism

C14 employs a unique carboxylate-based Ni-immobilization mechanism, distinct from conventional MICP and MIPP [50,51,52]. Fractionation experiments demonstrated that secreted metabolites (not cell surface adsorption) drive Ni removal: cell-free fermentation broth achieved 65.3 ± 2.8% Ni^2+^ removal, significantly higher than washed cells (28.7 ± 2.1%; Figure 4), indicating carboxyl-containing amino-acid metabolites as key functional components. XPS spectra confirmed coordination between Ni^2+^ (856.3 eV and 873.8 eV in Ni2p) and bacterial carboxyl groups (C=O peaks at 288.1 eV in C1s, 531.2 eV in O1s; Figure 5b,e). XRD patterns showed broad amorphous peaks (2θ = 23.18°), excluding crystalline Ni compounds (e.g., Ni(OH)_2_ and NiCO_3_). Combined with elemental analysis, precipitates were identified as stable amorphous nickel–carboxylate complexes (proposed formula: R-C_2_H_3_O_2_NNi), which require no exogenous chemicals and maintain stability across pH 4–10, overcoming the high costs and secondary pollution risks of traditional methods [53,54].

### 4.3. Integrated Plant-Growth-Promoting Mechanisms Under Nickel Stress

*Microbacterium algeriense* C14 enhances *Zinnia elegans* performance under nickel stress through synergistic multi-level mechanisms, consistent with emerging models of PGPR-mediated heavy-metal-stress alleviation [55].

Direct Metal Immobilization: Reduces soil-available nickel by 52.7% and plant nickel uptake by 20–35% via carboxylate precipitation, directly alleviating metal toxicity. This mechanism aligns with recent findings showing that PGPR can secrete low-molecular-weight organic acids (LMWOAs) that chelate and precipitate heavy metals in the rhizosphere [56,57].

IAA-Mediated Growth Promotion: Stimulates root development (up to 37% increase in root length) and shoot growth, enhancing nutrient and water acquisition, even under severe stress. This hormonal regulation is a well-documented PGPR mechanism for enhancing plant stress tolerance [40]. Elbagory et al. reported that IAA-producing Bacillus strains increased root biomass by 32–45% in nickel-stressed faba beans, facilitating better nutrient acquisition [58].

pH Modulation: Increases rhizosphere pH by 0.16–0.33 units, indirectly reducing nickel solubility and improving the activity of pH-sensitive soil enzymes. This pH-mediated mechanism has been increasingly recognized as a critical factor in PGPR-assisted phytoremediation [59]. Chen et al. demonstrated that nickel-resistant endophytic bacteria increased rhizosphere pH by 0.4–0.7 units, significantly reducing nickel availability and plant uptake in *Tamarix chinensis* [60].

Enhanced Antioxidant Defense: Elevates SOD activity by up to 36.43% and POD activity by 43.11%, effectively scavenging ROS and reducing lipid peroxidation. These results are consistent with numerous studies demonstrating that PGPR inoculation enhances antioxidant-enzyme activities in metal-stressed plants [61,62,63]. Jan et al. (2019) reported that metal-resistant endophytic bacteria increased SOD and POD activities by 58% and 52% [64].

Soil Quality Improvement: Enhances urease and acid phosphatase activities by 7.22–10.40% and 6.34–9.74%, respectively, at moderate nickel concentrations (N2–N3 groups), promoting soil nutrient cycling. This is a critical yet often overlooked benefit of PGPR inoculation in contaminated soils, where heavy metals typically inhibit microbial-enzyme activities [65,66]. Zhu et al. recently demonstrated that microbial consortia of phosphate-solubilizing and urease-producing bacteria not only stabilized heavy metals but also maintained soil-enzyme activities essential for nutrient availability [67].

### 4.4. Soil Environment Modification and Microbiome Shifts

C14 inoculation significantly improves soil environmental quality under nickel stress. It increases soil pH, reduces available nickel content, and optimizes the soil enzyme system—elevating nutrient cycling-related enzyme activities while moderately adjusting β-glucosidase activity. These soil-level changes are critical for creating a more favorable rhizosphere microenvironment that supports both plant growth and beneficial microbial activities [68].

Preliminary 16S rRNA sequencing reveals that C14 induces rhizosphere microbiome restructuring: under high nickel stress, it enriches Proteobacteria (particularly Gammaproteobacteria, accounting for 35.7% of the community) and reduces overall microbial diversity. Despite the reduction in diversity, key functional genera (e.g., Sphingomonas and Acidothermus) persist, maintaining essential soil functions through functional redundancy [69]. These shifts suggest potential microbiome-mediated synergistic effects that enhance remediation efficiency, though further replicated studies are needed for validation.

However, it is important to note that our microbiome analysis is preliminary, based on single or limited sampling points, and lacks the biological replication necessary for robust statistical validation. Further replicated studies incorporating temporal dynamics, spatial heterogeneity, and functional metagenomics are essential to confirm these observed patterns and elucidate the mechanistic basis of C14-microbiome-plant interactions [70]. Future research should also investigate whether the observed microbiome changes are stable over time or represent transient responses to C14 introduction, and whether they translate into improved long-term phytoremediation performance.

### 4.5. Practical Applications and Limitations

*M. algeriense* C14 demonstrates promising characteristics for PGPR-assisted phytoremediation of high-concentration nickel-contaminated sites. Several lines of evidence from this study collectively support this conclusion: (1) Strain C14 exhibits exceptional intrinsic nickel tolerance, maintaining viable growth at Ni^2+^ concentrations up to 800 mg·L^−1^ in liquid culture and surviving at 1200 mg·L^−1^, confirming its physiological adaptability to highly contaminated environments. (2) The novel carboxylate-based immobilization mechanism operates effectively without exogenous chemical supplements, reducing soil-available nickel by 14.4%, even at the highest contamination level (600 mg·kg^−1^ soil), demonstrating that the mechanism’s efficacy scales with contamination severity. (3) C14 inoculation sustained plant-growth-promoting effects under severe stress—at 600 mg·kg^−1^ soil Ni, inoculated plants showed 21.3% higher aboveground fresh weight and 19.8% higher root fresh weight compared to non-inoculated controls, while reducing plant Ni uptake by 38.7% (seedlings) and 49.9% (mature plants). (4) The strain maintained 47% of its IAA-producing capacity at 800 mg·L^−1^ Ni^2+^, ensuring continued growth promotion under extreme conditions. These integrated results—spanning bacterial physiology, soil chemistry, and plant performance—provide mechanistic and quantitative support for C14’s applicability in high-concentration nickel-contaminated sites.

## 5. Conclusions

This study successfully isolated and characterized *Microbacterium algeriense* C14, a multifunctional nickel-resistant PGPR, from the rhizosphere of *Zinnia elegans* in nickel-contaminated soil. As a valuable addition to the limited repertoire of nickel-resistant PGPR, C14 exhibits exceptional traits—it tolerates nickel concentrations up to 800 mg L^−1^, retains 47% of its IAA-producing capacity even under this severe stress, and can autonomously regulate the environmental pH to an optimal range of 8.3–8.7—demonstrating strong adaptability to diverse soil conditions.

A core highlight of this study is C14’s novel nickel-immobilization mechanism, distinct from conventional microbial-induced carbonate precipitation (MICP) or phosphate precipitation (MIPP). Confirmed by XPS and XRD analyses, C14 secretes carboxyl-containing metabolites that coordinate with Ni^2+^ to form stable amorphous nickel–carboxylate complexes. This mechanism requires no exogenous chemical supplements and maintains stability across a broad pH range, overcoming key limitations of traditional remediation technologies, such as high costs and secondary environmental risks.

Inoculation with C14 significantly enhances *Zinnia elegans* performance under nickel stress across all developmental stages: the germination index increases by up to 47.3%, seedling root length by 36.9%, and mature plant aboveground fresh weight by 21.32%. Simultaneously, it reduces plant nickel uptake by 38.7% (seedlings) and 49.9% (mature plants)—with mature shoot nickel content decreasing from 418.17 mg·kg^−1^ to 209.18 mg·kg^−1^ at 600 mg·kg^−1^ soil Ni^2+^—while increasing total soil nickel retention by 15.6% and decreasing DTPA-extractable available nickel by 14.4%, effectively mitigating metal toxicity and environmental mobility.

C14 also improves plant stress tolerance and soil quality: inoculated plants show enhanced antioxidant-enzyme activities (SOD increased by up to 36.43%; POD by 43.11%) and soluble protein content, to alleviate oxidative damage. Soil pH is elevated by 0.16–0.33 units, and urease and acid phosphatase activities are enhanced by 7.22–10.4% and 6.34–9.74%, promoting soil nutrient cycling and biological function.

Preliminary 16S rRNA sequencing of representative samples (*n* = 1 per treatment) reveals notable shifts in the rhizosphere microbial community under high nickel stress: C14 inoculation enriches Proteobacteria (particularly Gammaproteobacteria, accounting for 35.72% of the community) and reduces overall diversity. While these observations require validation with proper replication, they suggest potential microbiome-mediated synergistic effects on remediation efficiency, providing directions for future research.

In conclusion, *M. algeriense* C14 integrates a novel carboxylate-based immobilization mechanism with multiple plant-growth-promoting functions, offering the advantages of lower costs, minimal soil disturbance, and no biomass-disposal requirements compared to conventional remediation approaches. When paired with the ornamental plant *Zinnia elegans*, it provides dual benefits of environmental cleanup and aesthetic enhancement, making it highly suitable for remediating nickel-contaminated sites (e.g., urban green spaces and industrial brownfields) under controlled conditions. Future research should focus on field validation, molecular-mechanism elucidation, and optimization of inoculation protocols to support practical application.

## Figures and Tables

**Table 1:** Effect of *Microbacterium algeriense* C14 on seed germination index of *Zinnia elegans* under nickel stress.

Treatment	Germination Energy	Germination Percentage	Germination Index	Vitality Index
CK	56.67 ± 3.33 BCDbcd	67.78 ± 5.09 CDEcd	7.58 ± 0.93 Ed	76.96 ± 7.44 FGef
N1	64.44 ± 3.85 ABab	77.78 ± 1.92 ABCab	9.49 ± 0.45 Dc	102.84 ± 3.60 DEd
N2	56.67 ± 6.67 BCDbcd	64.44 ± 5.09 DEFcde	9.46 ± 0.17 Dc	88.07 ± 4.12 EFe
N3	48.89 ± 6.94 CDEdef	63.33 ± 5.77 DEFde	9.28 ± 0.47 DEc	73.59 ± 10.52 FGf
N4	53.33 ± 3.33 BCDEcde	58.89 ± 5.09 EFef	10.00 ± 1.63 CDc	58.30 ± 14.49 Gg
N5	43.33 ± 3.33 Ef	47.78 ± 5.09 Gg	8.80 ± 1.17 DEcd	30.77 ± 5.92 Hh
CKJ	64.44 ± 1.92 ABab	80.00 ± 3.33 ABa	11.58 ± 0.16 BCb	125.4 ± 2.09 BCc
N1J	72.22 ± 3.85 Aa	83.33 ± 3.33 Aa	13.98 ± 0.63 Aa	162.79 ± 12.86 Aa
N2J	57.78 ± 5.09 BCDbcd	72.22 ± 5.09 BCDbc	13.84 ± 0.77 Aa	140.35 ± 3.73 Bb
N3J	54.44 ± 8.39 BCDEcde	67.78 ± 5.09 CDEcd	12.86 ± 0.25 ABab	113.81 ± 5.26 CDcd
N4J	58.89 ± 5.09 BCbc	64.44 ± 1.92 DEFcde	12.97 ± 0.53 ABa	80.00 ± 7.22 Fef
N5J	45.56 ± 1.92 DEef	55.56 ± 1.92 FGf	9.63 ± 0.43 Dc	36.74 ± 5.88 Hh

Note: The data in the table are means and standard deviations; different lowercase letters in the same column indicate significant differences (*p* < 0.05), while different uppercase letters in the same column indicate extremely significant differences (*p* < 0.01).

**Table 2:** Effect of nickel stress *Microbacterium algeriense* C14 on growth index of *Zinnia elegans* seedlings.

Treatment	Stem Length (cm)	Root Length (cm)	Root Surface Area (cm^2^)	Root Projection Area (cm^2^)	Root Volume (cm^3^)
CK	5.252 ± 0.157 BCab	4.922 ± 0.382 Aa	0.663 ± 0.027 ABab	0.267 ± 0.031 ABbc	0.011 ± 0.001 ABCa
N1	6.041 ± 0.499 ABab	4.819 ± 0.537 ABa	0.666 ± 0.054 ABab	0.239 ± 0.016 Bcd	0.011 ± 0.002 ABa
N2	5.225 ± 0.396 BCab	4.089 ± 0.607 BCb	0.591 ± 0.104 Bb	0.228 ± 0.022 Bd	0.009 ± 0.001 BCDbc
N3	5.017 ± 0.678 BCDcd	2.887 ± 0.041 Dc	0.318 ± 0.054 Ccd	0.157 ± 0.020 Ce	0.007 ± 0.001 DEFcd
N4	4.087 ± 0.376 DEe	1.687 ± 0.315 Ed	0.251 ± 0.043 CDde	0.087 ± 0.010 DEf	0.005 ± 0.001 FGHde
N5	3.028 ± 0.651 Ff	0.502 ± 0.169 Fe	0.160 ± 0.039 Df	0.034 ± 0.007 Fg	0.002 ± 0.001 Hf
CKJ	5.686 ± 0.184 ABab	5.140 ± 0.287 Aa	0.698 ± 0.033 ABa	0.300 ± 0.028 Aa	0.012 ± 0.002 Aa
N1J	6.519 ± 0.514 Aa	5.124 ± 0.322 Aa	0.714 ± 0.025 Aa	0.286 ± 0.012 Aab	0.012 ± 0.002 Aa
N2J	5.609 ± 0.315 ABab	4.546 ± 0.235 ABab	0.677 ± 0.016 ABa	0.263 ± 0.010 ABbc	0.01 ± 0.001 ABCab
N3J	5.494 ± 0.202 ABab	3.354 ± 0.097 CDc	0.344 ± 0.030 Cc	0.167 ± 0.010 Ce	0.008 ± 0.001 CDEbc
N4J	4.322 ± 0.293 CDde	1.845 ± 0.190 Ed	0.269 ± 0.031 CDcd	0.095 ± 0.008 Df	0.005 ± 0.001 EFGd
N5J	3.129 ± 0.508 EFf	0.687 ± 0.171 Fe	0.185 ± 0.041 Def	0.049 ± 0.007 EFg	0.003 ± 0.001 GHef

Note: The data in the table are means and standard deviations; different lowercase letters in the same column indicate significant differences (*p* < 0.05), while different uppercase letters in the same column indicate extremely significant differences (*p* < 0.01).

**Table 3:** Effects of adding *Microbacterium algeriense* C14 under nickel stress on growth indices of *Zinnia elegans* mature plants.

Treatment	Plant Height (cm)	Stem Width (mm)	Aboveground Fresh Weight (g)	Underground Fresh Weight (g)	Root–Shoot Ratio
CK	44.14 ± 2.06 BCDcd	4.53 ± 0.10 Cc	2.49 ± 0.04 DEe	3.54 ± 0.10 Bb	1.42 ± 0.03 Aa
N_1_	46.73 ± 1.15 ABab	4.66 ± 0.13 BCbc	2.69 ± 0.06 BCDbcd	3.5 ± 0.03 Bb	1.30 ± 0.02 BCbc
N_2_	42.09 ± 1.54 CDde	4.24 ± 0.09 Dd	2.61 ± 0.07 CDcde	3.14 ± 0.06 Dd	1.20 ± 0.02 CDd
N_3_	37.84 ± 1.53 Ef	3.66 ± 0.09 Ff	2.32 ± 0.12 Ef	2.43 ± 0.06 Ff	1.05 ± 0.06 EFe
N_4_	32.51 ± 2.00 Fg	3.24 ± 0.11 Gh	1.79 ± 0.13 Gh	1.64 ± 0.05 Gh	0.92 ± 0.06 Gf
N_5_	26.58 ± 1.70 Gi	2.57 ± 0.08 Ij	1.36 ± 0.10 Hi	1.11 ± 0.04 Ij	0.82 ± 0.07 Gg
CKJ	46.71 ± 1.22 ABab	4.81 ± 0.04 ABab	2.75 ± 0.06 BCbc	3.79 ± 0.07 Aa	1.38 ± 0.01 ABab
N_1_J	48.47 ± 0.95 Aa	4.93 ± 0.07 Aa	2.99 ± 0.04 Aa	3.7 ± 0.03 Aa	1.24 ± 0.03 CDcd
N_2_J	44.98 ± 1.2 BCbc	4.50 ± 0.12 Cc	2.85 ± 0.07 ABab	3.33 ± 0.07 Cc	1.17 ± 0.05 DEd
N_3_J	41.49 ± 0.90 De	3.95 ± 0.07 Ee	2.57 ± 0.1 CDde	2.67 ± 0.09 Ee	1.04 ± 0.05 Fe
N_4_J	35.61 ± 1.25 EFf	3.49 ± 0.07 Fg	2.03 ± 0.11 Fg	1.79 ± 0.05 Gg	0.88 ± 0.08 Gfg
N_5_J	29.14 ± 0.60 Gh	2.85 ± 0.11 Hi	1.65 ± 0.11 Gh	1.33 ± 0.08 Hi	0.81 ± 0.09 Gg

Note: The data in the table are means and standard deviations; different lowercase letters in the same column indicate significant differences (*p* < 0.05), while different uppercase letters in the same column indicate extremely significant differences (*p* < 0.01).

**Table 4:** Different indices of microbial communities’ richness and diversity in different samples.

Treatment	Goods_Coverage	Observed_Species	Chao1	Shannon	Simpson
CK	0.98	3463	4180.41	9.45	0.99
N1	0.99	2454	2847.56	8.89	0.99
N2	0.99	2287.9	2761.53	8.64	0.99
N3	0.98	3984.9	4493.66	9.89	1.00
CKJ	0.99	1711	2103.81	8.08	0.99
N1J	0.99	2690	2987.7	9.46	1.00
N2J	0.99	2544	2728.67	9.27	0.99
N3J	0.99	1068.9	1352.57	6.90	0.98

## Data Availability

The 16S rRNA gene sequence of *Microbacterium algeriense* strain C14 has been deposited in GenBank under accession number: Px488027. All other data supporting the findings of this study are available within the article and from the corresponding author upon reasonable request.
